# Runx transcription factors in neuronal development

**DOI:** 10.1186/1749-8104-3-20

**Published:** 2008-08-26

**Authors:** Ken-ichi Inoue, Takashi Shiga, Yoshiaki Ito

**Affiliations:** 1 Department of Medical Biochemistry, Aarhus University, DK-8000C, Aarhus, Denmark; 2 Graduate School of Comprehensive Human Sciences, University of Tsukuba, 1-1-1 Tennodai, Tsukuba, 305-8577, Japan; 3 Institute of Molecular and Cell Biology, Biopolis Drive Proteos, 138673, Singapore

## Abstract

Runt-related (Runx) transcription factors control diverse aspects of embryonic development and are responsible for the pathogenesis of many human diseases. In recent years, the functions of this transcription factor family in the nervous system have just begun to be understood. In dorsal root ganglion neurons, Runx1 and Runx3 play pivotal roles in the development of nociceptive and proprioceptive sensory neurons, respectively. Runx appears to control the transcriptional regulation of neurotrophin receptors, numerous ion channels and neuropeptides. As a consequence, Runx contributes to diverse aspects of the sensory system in higher vertebrates. In this review, we summarize recent progress in determining the role of Runx in neuronal development.

## History

*Runt related *(*Runx*) genes are evolutionarily conserved developmental regulators in metazoa, where they play diverse roles in several different biological systems, including cell differentiation. One of the *Drosophila *pair-rule genes, *Runt*, controls segmentation, sex-determination and neuronal development [1]. The mammalian *Runx *gene was first identified as *AML1*, which is frequently involved in the chromosomal translocations associated with acute myeloid leukaemia (AML) [2]. Both *Runt *and *AML1 *encode a DNA binding subunit of the heterodimeric transcription factor PEBP2/CBF. Polyomavirus enhancer binding complex (PEBP2/PEA2) was identified during the characterization of the cellular mechanisms involved in differentiation using embryonal carcinoma cells [3]. CBF was first identified as a protein that binds to the core sequence of the murine retrovirus enhancer, which influences the tissue specificity of viral replication [4].

There are three mammalian *RUNX *genes, *RUNX1 *(*AML1*), *RUNX2 *(*CBFA1*) and *RUNX3 *[5]. *RUNX1 *is essential for definitive hematopoiesis and frequently involved in human leukaemia [6]. Runx2 is a master regulator of bone development [7]. Moreover, haploinsufficiency of *RUNX2 *is one of the causes of the hereditary bone disease Cleidcranial displasia [8]. *RUNX3*, the third member of the *RUNX *gene family, was the least characterized until gene targeting studies opened up new avenues of investigation into Runx function. First of all, *RUNX3 *is involved in many types of human cancer as a tumour suppressor [9,10]. Hypermethylation of the *RUNX3 *promoter and deletion of the *RUNX3 *gene are frequently observed in several cancers, and RUNX3 protein is now best considered as an apoptosis inducer [11,12]. Second, RUNX3 controls the generation of the T-cell sub-lineage [13-15]. In particular, transcriptional regulation of *CD4 *silencer and *Th-POK *have been described in detail [13,15]. Finally, Runx3 controls the development of proprioceptive dorsal root ganglion (DRG) neurons [16,17]. The last discovery was particularly relevant to developmental neurobiology and, since then, several groups have characterized not only Runx3, but also Runx1 as a crucial regulator of DRG neurogenesis [18,19].

### Expression of Runx1 and Runx3 in the nervous system

Earlier *in situ *hybridization studies indicated strong expression of *Runx1 *mRNA in spinal motor neurons, DRG, cranial ganglia and specialized sensory epithelial structures such as olfactory and gustatory mucosa, and follicles of the vibrissae [20]. Subsequently, the generation of specific antibodies against Runx1 and Runx3 and the utilization of *Runx1*^*β-gal *^or *Runx3*^*β-gal *^mice revealed the expression of Runx1 and Runx3 in the nervous system in more detail [16,21,22]. Runx1 is synthesized in both the central and peripheral nervous systems of mouse embryos. In the central nervous system, Runx1 is synthesized in selective populations of somatic motor neurons in the spinal cord and in cholinergic branchial and visceral motor neurons in the hindbrain, such as dorsal vagal nucleus and nucleus ambiguus [21,22]. In the peripheral nervous system, Runx1 is localized to DRG and selective cranial ganglia, including trigeminal (V) and vestibulocochlear (VIII) ganglia and the glossopharyngeal-vagal (IX-X) ganglia complex [21,22]. In contrast to Runx1, Runx3 is confined to the peripheral nervous system, specifically to DRG and cranial ganglia [16,21]. Although Runx1 and Runx3 are almost exclusively found in postmitotic neurons in the central nervous system and peripheral ganglia [16,21,22], a rare exception is the expression of Runx1 in proliferating progenitors of the olfactory epithelium [23]. These observations suggest Runx1 and Runx3 have extensive functions in the mammalian nervous system.

### Roles of Runx3 in the development of DRG neurons

**Figure 1:**
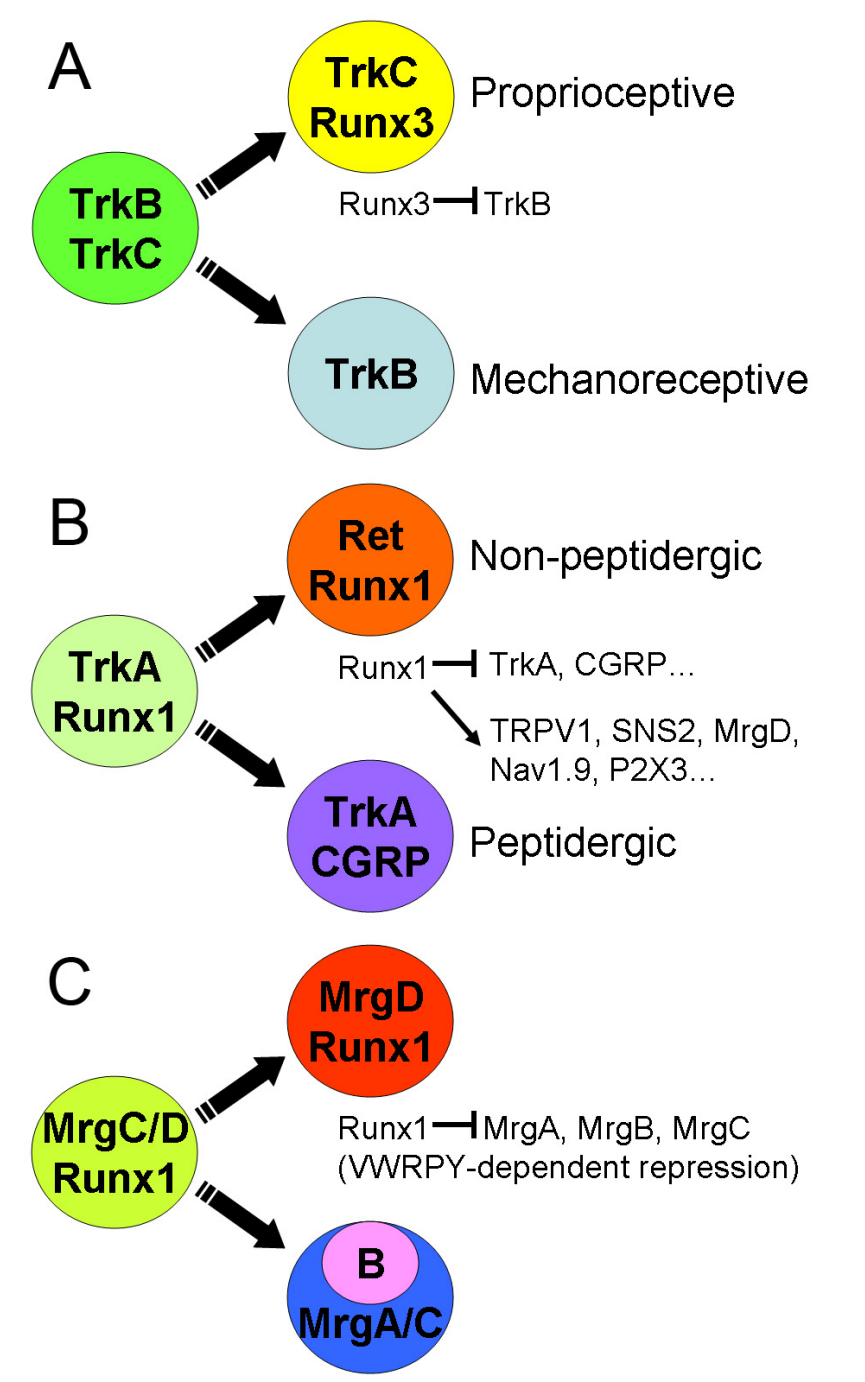
**Runx proteins control the diversification of sensory neurons.(a) **Proprioceptive (TrkC^+^) and mechanoreceptive (TrkB^+^) DRG neurons are derived from the common precursors (TrkB^+^, TrkC^+^). During segregation of two complementary sensory populations, Runx3 represses *trkB *expression in TrkC^+ ^neurons. **(b) **During early postnatal periods, TrkA^+ ^DRG neurons differentiate into two nociceptive subpopulations; TrkA^+ ^peptidergic neurons, and Ret^+ ^non-peptidergic neurons that repress *trkA*. In Ret^+ ^non-peptidergic neurons, Runx1 represses *trkA *and neuropeptide *CGRP*. Runx1 also activates a number of nociceptor-specific G protein coupled receptors, ATP channels, and TRPV channels. **(c) **G protein coupled receptor MrgA, B and C are under dynamic transcriptional regulation in DRG neurons. A carboxy-terminal VWRPY motif of Runx proteins is critical for binding to Groucho corepressor. Runx1, which lacks VWRPY, fails to repress MrgA, B and C in DRG neurons.

DRG neurons convey peripheral somatosensory stimuli to the spinal cord. There are three major subpopulations of DRG neurons – nociceptive, mechanoreceptive, and proprioceptive – which differ in their cell size, dependency on neurotrophins, and distinct axonal terminal fields in the spinal cord and peripheral tissues. Runx1 and Runx3 are synthesized initially in TrkA^+ ^nociceptive and TrkC^+ ^proprioceptive neurons, respectively (Figure 1) [17,24,25]. This complementary expression pattern suggests specific roles for Runx1 and Runx3 in subtypes of DRG neurons. Indeed, the phenotype of Runx3 knockout mice is similar to that of NT3 and TrkC knockout mice [16,17,26-29]. Namely, Ia/Ib type DRG neurons fail to form a stretch reflex circuit with motor neurons in the spinal cord, resulting in severe motor discoordination [16,17]. What is the molecular basis of the phenotype? Several elegant studies have been performed to answer this question.

First, the role of Runx3 in the neurotrophin receptor phenotype was shown by Arber and her colleagues [25], who thoroughly compared neurotrophin receptor synthesis in mouse strains in which *Runx3 *had been disrupted or expressed ectopically. In DRG neurogenesis, dynamic changes are observed during the synthesis of neurotrophin receptors (TrkB, TrkC) [25]. At early developmental stages, most DRG neurons synthesize TrkC protein first before the onset of TrkB synthesis. Thus, some TrkC^+ ^DRG neurons co-synthesize TrkB (Figure 1a). Subsequently, the ratio of TrkB/TrkC-hybrid neurons declines to produce DRG neurons that synthesize either TrkC or TrkB (Figure 1a). During this segregation, Runx3 is observed in most TrkC^+ ^neurons but not in TrkB^+ ^neurons [25]. One of the functions of Runx3 is to repress TrkB when DRG neurons acquire TrkC^+ ^identity (Figure 1a) [25].

Second, the axonal outgrowth and/or axonal guidance of propiroceptive DRG neurons are also regulated by Runx3. Two different interpretations were proposed for the phenotype of the *Runx3*-/- DRG. One group proposed that Runx3 controls the appropriate axon targeting of *trkC*-expressing proprioceptive DRG neurons to motor neurons [16]. However, another group observed massive cell death of TrkC^+ ^neurons in *Runx3*-/- DRG in apparent contradiction to the previous proposition [17]. A recent study with *Runx3 *and *Bax*-double knockout mouse revealed clearly that the axonal projection of propioceptive DRG neurons to motor neurons is still lost in the *Runx3 *mutant even in the absence of apoptosis [30]. The study further clarified that the initial model 'Runx3 → TrkC and Runx1 → TrkA' might not apply to later developmental stages [30]. They observed that Runx3 co-localizes not only with TrkC, but also TrkA and TrkB at postnatal day 0 (P0) [30]. Of note, Runx1^+ ^and Runx3^+ ^neurons were clearly segregated at embryonic day 16.5 (E16.5) but almost all Runx3^+ ^neurons co-synthesize Runx1 at E18.5 and P0 [30]. It is possible that Runx3 has some functions not only in proprioceptive neurons, but also in nociceptive neurons [30]. Overall, the evidence obtained from *Runx3 *and *Bax *compound mutants support a role for Runx3 in the control of axonal projection, although the molecular mechanisms remain unknown [30]. Prior studies showed that DRG explants from *Runx3*-knockout mouse embryos extended short neurites in the presence of NT3, a ligand for TrkC, but not in the presence of NGF, a ligand for TrkA [16]. This suggests that Runx3 may regulate the axonal outgrowth of specific DRG neurons independently of the target tissue. On the other hand, Chen *et al*. [24] revealed, using a *tour de force *method, that Runx3 activity determines the dorso-ventral position of axonal termination of DRG neurons in the spinal cord. DRG neurons with high Runx3 activity extended their axons far into the ventral spinal cord like proprioceptive neurons, whereas those neurons with low Runx3 activity extended their axons into the dorsal spinal cord. Ectopic expression of Runx3 is sufficient to drive axons from the dorsal to the ventral spinal cord, indicating that Runx3 *per se *has instructive roles in central axon targeting in DRG neurons.

Thus, Runx3 controls the neurotrophin receptor phenotype as well as the axonal projection of proprioceptive DRG neurons. The two functions may not be mutually exclusive but closely related to each other. For example, NGF/TrkA signalling and NT3/TrkC signalling are required for proper axonal projection [31,32].

### Roles of Runx1 in the development of DRG neurons

In contrast to Runx3, the study of Runx1 function in DRG development was delayed owing to the early embryonic lethality of the targeting mouse [22,23,33]. Thus, Runx1 knockout mice die due to a lack of definitive hematopoiesis by E12.5, which is before the onset of major events in the development of TrkA^+ ^DRG neurons. However, recent studies have investigated the roles of Runx1 in DRG neurons using different experimental models.

First of all, Runx1 controls the lineage diversification of nociceptive neurons [25,33,34]. During late embryonic and early postnatal periods, *trkA*-expressing neurons differentiate into two subpopulations of nociceptive neurons; *trkA*-retaining peptidergic neurons, and non-peptidergic neurons that repress *trkA *and instead activate *Ret*, a receptor for glial-derived neurotrophic factor (GDNF; Figure 1b). During the late embryonic stages, most *trkA*-expressing DRG neurons coexpress *Runx1 *(Figure 1b). Postnatally, Runx1 disappears in *trkA*-retaining peptidergic neurons but continues to exist in *Ret*-inducing non-peptidergic neurons (Figure 1b). Using the *Runx1*-conditional knockout mouse, it was shown that Runx1 is dispensable for the *de novo *induction of TrkA [34]. This was confirmed by Shiga and his colleagues [33], who used a different gene-targeting method that relied on the rescuing of *Runx1 *expression in hematopoietic cells. However, Marmigere *et al*. [35] showed that virally expressed *Runx1 *induced *de novo *synthesis of TrkA in the DRG and spinal cord of chick embryos. One possible explanation is that the minimal enhancer of *trkA*, which Runx1 regulates [35], may not be required for the *de novo *induction of *trkA *expression [36]. On the other hand, Runx1 is essential for the late repression of *trkA *and induction of *Ret *when TrkA^+ ^and Ret^+ ^neurons segregate (Figure 1b) [34]. In addition to *trkA*, Runx1 also represses the neuropeptide, calcitonin-gene-related peptide (CGRP; Figure 1b) [25,33,34]. More surprisingly, nearly all the known marker genes for nociception are under the control of Runx1. In the conditional *Runx1 *mutant DRG, expression of a number of nociceptor-specific G protein coupled receptors, ATP channels, and TRPV channels is attenuated (Figure 1b) [34].

Similar to Runx3, Runx1 also regulates the axonal outgrowth and guidance of nociceptive neurons. Marmigere *et al*. [35] revealed that the transfection of Runx1 into boundary cap-derived neural crest stem cells increased neurite length and branching. In Runx1-knockout mice, the axonal projection to laminae IIi of the dorsal spinal cord was perturbed [33,34]. In the wild type, peptidergic nociceptive axons project to layer I/IIo in the superficial dorsal horn, whereas non-peptidergic nociceptive axons project to deeper layer IIi. In Runx1-knockout mouse, non-peptidergic axonal projection displays dorsal shift to layer I/IIo [34].

Thus, Runx1 controls a battery of genes that are associated with the generation of non-peptidergic nociceptive neurons. The findings that both Runx3 and Runx1 play critical roles in distinct sensory neurons suggest that Runx factors are involved in the evolution of sophisticated sensory systems in higher vertebrates.

### Upstream/downstream genes

The upstream signals and transcriptional regulation of *RUNX *genes have been studied in non-neuronal tissues [37]. However, only limited studies have addressed this issue in the nervous system. Both *Runx3 *and *Runx1 *genes contain Brn-3a binding sites in their 5'-upstream regions, suggesting that *Runx3 *and *Runx1 *are candidate downstream targets of Brn-3a, a well characterized transcription factor in sensory neurons [38,39]. Microarray studies have shown decreased levels of *Runx1 *and *Runx3 *transcripts in the sensory neurons of *Brn-3a*-knokout mice [40,41]. Kramer *et al*. [25] investigated the putative upstream signal of *Runx1*/*Runx3 *in DRG neurons. Plausible candidates are TrkC/TrkA signalling and the basic helix-loop-helix transcription factors Ngn2/Ngn1; however, a genetic study has excluded these possibilities [25]. Ginty and colleagues [42] investigated the roles of NGF and the Ret receptor in DRG neurons. In *Ngf-Bax *compound knockout DRG, TrkA neurons are hypotrophic although *de novo Runx1 *expression is unaffected [42]. However, *Runx1 *expression is not maintained to the neonate stage and the expression of all putative Runx1 target genes is altered [42]. Thus, NGF signalling is essential for sustained expression of *Runx1*. In *Ret *conditional knockout DRG, *Runx1 *expression is normal but a part of Runx1 target genes are affected, suggesting the GFR/Ret dependent transcriptional regulation by Runx1 in DRG neurons [42]. Although this study placed Runx in a pivotal position in developmental signalling cascades, the upstream signalling event(s) still remains elusive.

On the other hand, how does Runx1/Runx3 regulate downstream transcriptional cascades? In DRG neurons, TrkC is a critical signalling receptor involved not only in the control of cell survival, but also in axon path-finding and fate determination of proprioceptive DRG neurons [32,43,44]. Therefore, it is natural to infer that *trkC *is a transcriptional target of Runx3 [17]. However, unbiased computational analysis suggested that a *cis*-regulatory element exists in the gene locus of TrkB, rather than in the gene locus of TrkC [45]. This was unexpected because *trkB *is expressed in neurons of an alternative sensory fate, TrkB^+^TrkC^- ^neurons [43]. The strategy "to repress alternative traits" appears to be a common feature in neuronal lineage commitment [46]. At the molecular level, *trkB *possesses a conserved cluster of Runx binding sites that function as a silencer of the *trkB *promoter in cultured DRG neurons [45]. In *Runx3 *knockout DRG, derepression of *trkB *seems to be a crucial event, influencing lineage commitment [25,45], and, eventually, resulting in drastic behavioural consequences [16,17].

Runx protein works both as an activator and repressor, depending on the molecular context [47]. The finding that Runx3 represses *trkB *raises a question as to the identities of its partner molecules in the transcriptional repressor complex. The function of Runx1 as a transcriptional repressor has been widely studied [48,49]. A plausible candidate in the context of DRG is the Groucho corepressor. In motoneuron fate specification, Groucho-mediated repression is a common mechanism for homeodomain proteins containing the EH1 domain [46]. Runx proteins have the evolutionarily conserved VWRPY carboxy-terminal motif, which is considered to be critical for Groucho binding/function [50,51]. Yarmus *et al*. [52] generated mice in which Runx3 lacks these amino acids. Surprisingly, VWRPY knockout mice displayed the normal development in DRG neurons, though they showed the phenocopy to *Runx3 *knockout mice in dendritic cells [52]. The results suggest that Runx3 represses *trkB *through a Groucho independent mechanism. Recently, Ma and his colleagues [53] investigated the significance of the VWRPY motif of Runx1 in DRG neurons. *Runx1 *cDNA, which lacks the VWRPY coding sequence, was knocked into the native *Runx1 *locus in *delta446 *mice [54]. In the *delta446 *mice, derepression of Mrg class G-protein-coupled receptor genes was observed, suggesting that Mrg genes are repressed by a Groucho-dependent mechanism (Figure 1c) [53]. Interestingly, two putative target genes that are repressed by Runx1, *trkA *and *CGRP*, were unaffected in the *delta446 *mice [53]. These results suggest that Runx1 represses target genes through either a Groucho-dependent or an independent mechanism in DRG neurons.

Chen *et al*. [34] indicated that Runx1 controls nearly all known marker genes critical for nociceptive functions. Such global control by Runx over the transcription landscape is also observed in other physiological functions, such as hematopoietic stem cell formation (Runx1) and osteoblast maturation (Runx2). How this unique transcription factor has such a huge influence on many different transcriptional cascades remains a challenging question.

### Other neurological phenotypes of Runx1/Runx3 knockout mice

Stifani and his colleagues [22,23] have worked on the neurological phenotypes of the Runx1 knockout mouse other than those arising from defects in DRG neurons. They analysed the cranial sensory neurons as well as cholinergic branchial and visceral motor neurons of hindbrain at an early embryonic stage [22]. The expression of Runx1 was restricted to post-mitotic neurons, and disruption of Runx1 resulted in massive neuronal apoptosis [22]. In contrast to this finding, *Runx1 *is expressed in the proliferating neuronal progenitors/precursors of olfactory receptor neurons (ORNs) [23]. Runx1 drives the cell cycle in ORN progenitors through transcription repression of the cyclin dependent kinase inhibitor *p21 *[23]. Unlike DRG, they did not observe any changes in the lineage markers in the neurons examined (cranial, hindbrain and olfactory), indicating that Runx1 has distinct functions in different types of neurons [22,23].

The study of the neurological function of Runx3 other than in DRG is very limited. Levanon *et al*. [17] reported that TrkC^+ ^neurons in the trigeminal ganglion survive in contrast to DRG neurons in *Runx3*-/- mouse. Most *Runx3 *knockout mice of the C57/B6 strain die within one day after birth [9,16]. The main cause of death may be starvation, as little milk is found in the stomachs of these mice [9]. As this is probably related to the pups being unable to swallow milk, it is interesting to note that *Runx3 *is strongly expressed in cranial ganglia, including the glossopharyngeal ganglion [16,17]. It is possible that Runx3 is essential for the functional glossopharyngeal system (swallowing), suggesting the critical roles in developmental cranial neurons.

## Conclusion

Although the roles of Runx in neural development have just begun to be investigated, studies in gene knockout mice indicate that the roles of Runx in the nervous system are as important as its roles in other, non-neuronal tissues. However, a number of open questions should be addressed in the future. First, upstream signalling cascades remain elusive. The mRNA expression and protein synthesis for *Runx1*/*Runx3 *are tightly regulated and DRG is one of the tissues in which *Runx1*/*Runx3 *display their highest protein levels among the entire body; how do DRG neurons achieve such a high protein level for *Runx1*/*Runx3*? Second, the molecular bases of tissue specificity are largely unknown. Runx1 and Runx3 are highly homologous but they control the development of distinct subpopulations of sensory neurons. In particular, Runx1^+ ^neurons and Runx3^+ ^neurons project axons into totally different target tissues; how is this specificity achieved? Third, transcriptional regulation is not the only determinant of DRG neurogenesis. Ectopic synthesis of TrkC receptor *per se *influences the lineage commitment of DRG neurons [44], while Runx3 plays a crucial role in TrkB/TrkC status [25,45]. It is likely that Runx and neurotrophin status are closely related to each other. How this cross-regulation is carried out is a challenging question. Finally, since all three Runx proteins have common features, some of the knowledge about Runx function in oncology, haematology, immunology and bone biology is likely to be applicable to neuroscience as well, particularly at the molecular level [53].

## Competing interests

The authors declare that they have no competing interests.

## Authors' contributions

The first draft of this review was written by KI together with TS, which was then complemented by YI. The figure was composed by KI.
